# Peer Helpers’ Experience of Participation in an Adventure-Based Experiential Learning Program: A Grit Perspective

**DOI:** 10.3389/fpsyg.2022.795824

**Published:** 2022-04-04

**Authors:** Marica Pienaar, Johan C. Potgieter, Cornelia Schreck, Ilana Coetzee

**Affiliations:** ^1^School for Psychosocial Health, North-West University, Potchefstroom, South Africa; ^2^Physical Activity, Sport, and Recreation, North-West University, Potchefstroom, South Africa; ^3^Student Counselling and Development, North-West University, Potchefstroom, South Africa

**Keywords:** peer helper, adventure, experiential learning, grit, qualitative analysis

## Abstract

The study focused on the adventure-based experiential learning (ABEL) component of the North-West University peer helper training program. The aim of this study was to explore and describe a group of peer helpers’ subjective experiences of their participation in an ABEL program, with a focus on how these experiences related to the concept of grit. A total of 26 students at the North-West University, both male and female, participated in the study. A qualitative research approach with a case study research design was used. The participants completed daily reflective diaries for the duration of the three-day ABEL program. After 3 months of performing their duties as peer helpers, the same individuals participated in focus group interviews. Themes were identified through inductive analysis and discussed regarding their relevance to the concept of grit. The main themes that emerged from both phases of data collection included intra-, inter-, and transpersonal/transcendent aspects, within which participants regularly referred to elements of grit. It was concluded that ABEL, due to its unique nature and demands, provides an ideal mechanism for the facilitation of personal growth on various levels. More specifically, through its clear association with the improvement and/or development of participants’ grit, it could equip these students to be more effective in their role as peer helpers.

## Introduction

Peer helper programs have increasingly been used by universities to address the extensive range of difficulties that especially first-year students’ experience. These programs have become a valuable resource that allows university counseling centers to serve more students (Lee and Bush, 2003; Lennox Terrion and Leonard, 2010). A peer helper is a registered student who has been selected, trained, and supervised to assist student counseling services in performing interpersonal helping tasks with persons of similar age or experience (De Jager, 2012). The most demanding period for fulfilling their duties is usually during the registration and orientation (R&O) program of the first-year students, where peer helpers ordinarily assist with providing optimal support with the various adjustment difficulties first-year students may experience.

Despite the significant value added by peer helper programs, De Jager (2012) reports a number of challenges in maintaining these programs in higher education. Peer helpers sometimes have excessive responsibilities placed on them. Because peer helping is a voluntary process, peer helpers’ commitment and continued motivation to perform their duties have become one of the main challenges. Research regarding the protective effect of various character strengths during times of adversity has however been receiving increasing attention, especially within the rapidly growing movement of positive psychology (Park et al., 2004; Gillham et al., 2011). The facilitation of psychological strengths among individuals who find themselves in challenging circumstances, such as these peer helpers, has been found to oppose the development of negative symptoms (Gillham et al., 2011; Niemiec, 2020). Niemiec (2020) added that character strengths play a central role during times of adversity, not only as a source of protection but it also offers a shift of mindset and helps to navigate and manage the struggles. Within the movement of positive psychology there are ongoing efforts to expand our understanding on the specific character strengths that may foster effective adaptation to adversity and assist individuals to effectively engage in important life activities (Buckingham and Richardson, 2021). As one of the most recent additions to this family of strengths, the possession of “grit” has shown itself to facilitate individuals’ ability to maintain focus on their original goals in the face of adversity (Duckworth and Quinn, 2009). Described as the character strength of passion and perseverance in the pursuit of long-term goals, even when encountering challenges or adversities (Duckworth et al., 2007; Hochanadel and Finamore, 2015), grit is generally accepted as a multidimensional construct that includes elements such as resilience, perseverance, and commitment (Robertson-Kraft and Duckworth, 2014). However, no single model that distinguishes all the different components of this fairly new phenomenon currently exists. Incantalupo-Kuhner (2015) suggested a strong causal and positive relationship between grit and resilience but reiterates that these are not identical constructs. Georgoulas-Sherry and Kelly (2019) concur by emphasizing the importance of distinguishing similar constructs, like resilience, grit, and hardiness from one another. These authors cite compelling evidence that these terms, although often used interchangeably, constitute constructs that are empirically different from each other. Although grit, for instance, is a dimension of resilience in successfully adapting to overwhelming stress and adversity, Perkins-Gough (2013) reiterates that the reason why some individuals succeed at persisting towards their goals, while others ultimately give up, is that grit represents not only showing resilience when facing setbacks, but also remaining loyal to commitments.

Within existing peer helper programs, incentives like stipends or food are most often used to maintain peer helpers’ commitment (De Jager and Ntlokwana, 2011). Showing grit however means possessing an intrinsic, future-orientated motivation to stay on one’s course in the long term, even in the absence of any tangible reward or clear indicators that the hard work will be rewarded in due course (Duckworth et al., 2007). Grit therefore redefines the extent of effort that individuals are willing to put into reaching their goals. The strong inclination towards remaining goal oriented, together with the capacity for psychological regulation, allows the gritty individual to persevere despite tedious and frustrating circumstances. As a result, it also provides them with the ability to cope with more challenging and tough circumstances (Duckworth et al., 2011; Ceschi et al., 2016; Duckworth et al., 2016). Eskreis-Winkler et al. (2014) cite several studies across multiple domains that offer examples where grit appears as a significant predictor of success and conclude that individuals with higher levels of grit consistently show fewer dropouts from their respective life commitments. Recent studies affirm that grit has also been associated with a number of positive outcomes, including lower suicidal ideation during stressful life events (Blalock et al., 2015), successful completion of military training that involves a high degree of mental and physical stress (Farina et al., 2019), improved mental and physical quality of life (Traino et al., 2019), as well as academic achievement among university students in the wake of the unprecedented and challenging circumstances imposed by the COVID-19 pandemic (Ghanizadeh, 2021). Considering these findings, it is argued that grit reinforces positive coping and reduces the perception of threat (Buckingham and Richardson, 2021). It therefore seems reasonable to hypothesize that for the peer helper, and ultimately the peer helper system to function optimally, grit can be considered a fundamental characteristic.

One of the most significant objectives of positive psychology is the development and nurturance of character strengths (Ghielen et al., 2018). In line with this, Ris (2015) believes that grit is not a fixed quality and opines that it can be developed among students through facing adversity outside of academic traditions. Adventure-based experiential learning (ABEL) provides unique possibilities in this regard. The challenging nature of adventure activities that typically form part of ABEL programs represent an opportunity for fostering growth and change. These programs typically incorporate a variety of challenging adventure activities as a means of facilitating the development of physical, social, and mental competencies, thereby strengthening positive psychological characteristics (Sheard and Golby, 2006; Lee and Ewert, 2013) both within individuals and groups. More specific to our research group, the adventure component of ABEL has been found to engage the motivation and interest of participants, and that it develops participants’ determination to persevere when situations become complicated (Sibthorp and Jostad, 2014). As Csikszentmihalyi and Csikszentmihalyi (1990, p.17) conclude, “challenge gives people vision and direction, focus, and perseverance…”—a statement that strongly refers to the concept of grit. Although no studies could be found that links ABEL to the development of grit specifically, existing research does therefore suggest that ABEL, due to its specific nature and demands, may be an ideal intervention for the facilitation of personal growth and, more specifically, the improvement and/or development of participants’ grit.

When it comes to peer helpers specifically, Aladağ and Tezer (2009) state that their success mainly depends on the self-growth and skills they develop through their training programs. ABEL, when included as part of this training, may therefore provide opportunities for the development of a variety of character strengths (Berman and Davis-Berman, 2005), including grit. The utilization of an ABEL program as a developmental intervention might be an efficient way for peer helpers to recognize and understand their own strengths, weaknesses, and personal resources, which could prove beneficial in terms of the numerous challenges they may face in successfully reaching their objectives (Sheard and Golby, 2006; Strydom et al., 2012). An improved understanding of the potential impact of such interventions on participants’ grit may prove valuable for the development of training programs aimed at the improved functioning of peer helpers at universities.

The aim of the current study was therefore to explore and describe the subjective experiences of a group of peer helpers’ participation in the ABEL program, with a specific focus on how these experiences related to the concept of grit.

## Materials and Methods

A holistic single-case, case-study design with a qualitative approach was used (Baxter and Jack, 2008). A case study is a bounded system and explores an existing phenomenon within its real-world context when the boundaries between the phenomenon and the context are not distinctly evident (Maree, 2007). It allows the researcher to focus on a “case” and hold onto a holistic perspective, while it aims at gaining greater insight and understanding of the dynamics of a specific situation (Maree, 2007; Yin, 2014). More specifically, the present study made use of a single-case (holistic) design, where different experiences are described within one group of participants finding themselves in the same context (Baxter and Jack, 2008). The researcher in this case concentrated only on the experiences of peer helpers of the NWU with respect to an aspect of their training program.

The peer helpers participated in a 3-day program that included the following activities: Upon arrival on the first day participants were divided into two groups. The one group received an introduction to whitewater paddling and participated in water games on the river with inflated rafts. At the same time the second group went rock climbing and abseiling on a 10-meter artificial abseiling wall. Groups were rotated after completion of these activities. The second day of the program involved a 15 km river rafting session, after which they did a 5 km hike into the mountain to their overnight site. Upon arrival participants had to set up camp and had to prepare their own food. On the last day, participants went on an early morning hike of about 8 km back to basecamp to conclude the program.

### Demographics and Recruitment of Participant Group

Peer helpers are undergraduate students registered with the NWU, residing in on-campus residences. The peer helpers form part of a well-defined group of university students, who all partake in the same training program to prepare them for their responsibilities as peer helpers. Since the participants hold a defining characteristic that is needed for the data of this study, purposive sampling (a form of non-random sampling) was used in the selection of the participants, with a specific purpose in mind (Maree, 2007). The main aim for purposive sampling is the selection of a small number of people whose information will generate an in-depth understanding of the people, program and situation involved (Yilmaz, 2013).

The participating peer helpers from the NWU (Potchefstroom site) were pre-selected by the staff of Student Counselling and Development (SCD) for the 2017 academic year. A pre-determined question and case-study based selection process were followed by interviewing three peer helpers per residence. One peer helper was appointed per residence based on observed and reported character traits, emotional adjustment, and peer relevance identified during the selection interviews. Permission for data to be collected from the participants, specifically regarding their experiences of the ABEL component of their training program, was obtained from the acting director of SCD—the principal gatekeeper of the peer helpers—as well as from the Dean of Students (now Director Student Life) at the NWU. Further discussions were held with the staff members involved in the training of the peer helpers (as secondary gatekeepers to the peer helpers), as well as the staff responsible for the ABEL program. The training included the total population of 31 peer helpers, both male and female. The final sample size consisted of 26 participants, 11 of which were male and 15 female, who all voluntarily agreed to participate in the research.

### Data Collection

Qualitative data regarding the individuals’ experience of their participation in the ABEL component of their training program was collected in two phases—during, as well as after their training had taken place. The first phase of data collection required the participants to complete daily reflective diaries for the duration of the 3-day ABEL program. The diaries provided some guidance on the reflective process to make it as easy as possible for the participants, without providing too much structure that may potentially restrict the nature of data obtained. The diary entry started with *“My experience of today…,”* with adequate space provided for the participants to reflect on their own experiences in writing. Two incomplete sentences followed: “M*y favorite part of today’s adventure activity*…” and “W*hat I have learned about myself*…”

The second phase of data collection commenced in the form of focus group interviews after peer helpers completed their training and had performed their duties as peer helpers for a period of three months. Morgan (2013) recommends that a focus group preferably consists of 6–10 individuals. Three focus group interviews were conducted, each of which lasted for approximately 60 min and consisted of 8–10 individuals. The questions of the focus group interviews were structured to obtain an informed understanding of the participants’ experiences as peer helpers, and their reflections on the value that their participation in the ABEL program added to their current roles as peer helpers. The opening question of the focus group interviews was: *“What were your experiences of participating in the adventure-based component of your training program?”* This opening question was followed by secondary questions, such as: *“What did you learn about yourself during the program?” “What value, do you feel, the adventure program added to your role as a peer helper?” “What experience in the last few months reminded you of your participation in the adventure program?”* and *“What personal growth do you feel might have resulted from your participation in this adventure program, if any?”* All interviews were audio-recorded and transcribed with the participants’ permission.

### Data Analysis

Thematic content analysis was conducted following an inductive approach. Thematic analysis can be described as the groundwork method of qualitative analysis, aimed at identifying and analyzing patterns in the content (Clarke and Braun, 2013). The main purpose of inductive analysis of the data is to allow research findings to emerge from the frequent, dominant, or significant themes and to develop concepts or a model inherent in raw data (Thomas, 2003; Maree, 2007). By using an inductive approach to thematic analysis, several steps were taken to analyze the data effectively (Clarke and Braun, 2013). Firstly, the researchers thoroughly familiarized themselves with the data from the diaries and focus group interviews after it had been transcribed. After immersing themselves in the transcriptions, they proceeded to generate initial codes by means of documenting where and how patterns occurred and reoccurred. Themes were then constructed from the patterns in the data relevant to the research question. After reviewing the themes, the researchers defined and further refined each theme and wrote a detailed analysis of each (Clarke and Braun, 2013). The data gathered from the participants’ reflective diaries during the ABEL program and data from the focus group interviews conducted after they have performed their duties as peer helpers for a few months, was analyzed separately.

The validity of the process of data gathering and analysis were ensured by following the criteria proposed by Lincoln and Guba (1985) and Korstjens and Moser (2018). Firstly, *credibility* was ensured by means of data triangulation, with the researchers gathering data on the same topic from a diverse group of participants, using both personal journals and focus group discussions. Investigator triangulation was also used, with both the primary investigator and the second author involved in coding and analyzing the data obtained. Secondly, *transferability* was addressed by providing as detailed a description of the participants, the research setting and the research process as possible, ensuring the reader’s ability to determine the relevance and transferability of research findings to his/her own setting. Thirdly, *dependability* (which refers to the stability of findings over time) was addressed through gathering data at two points in time, both during and after completion of the ABEL intervention. *Confirmability* was ensured by establishing a clear audit trail, which describes in detail the research steps taken from the start of the project to the reporting of the findings. Lastly, *reflexivity* was ensured by the primary researcher using a diary to document and examine her assumptions, values, and preconceptions.

### Ethical Clearance

Before the participants were invited to participate in the research study, ethical approval was obtained from the Health Research Ethics Committee (HREC) of the NWU (ethics approval number NWU-00361-16-A1). Throughout the entirety of the study, the researcher adhered to the ethical guidelines of the North-West University’s Health Research Ethics Committee (NWU-HREC) as well as the Health Professions Council of South Africa (HPCSA: Health Professions Act 56 of 1974).

## Results

**FIGURE 1:**
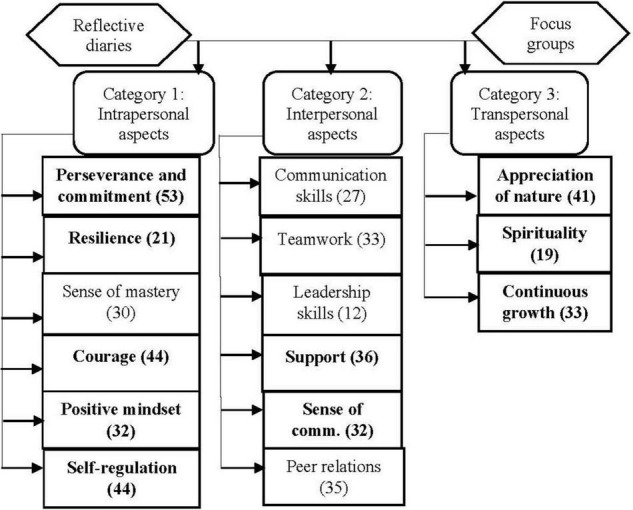
Themes identified regarding peer helpers’ experience of participation in adventure-based experiential learning (ABEL).

Following the completion of each phase of data collection, the themes and subthemes that emerged were found to represent three broad categories, which involved *intra-, inter-, and transpersonal/transcendent* aspects of participants’ functioning. These categories were subsequently used to organize and report the emergent themes, which are graphically depicted in Figure 1. The frequency with which each of the themes were mentioned during the diary entries and focus group discussions are indicated between brackets. Due to the significant degree of overlap between the themes that emerged from the two phases of data collection, the findings will be reported in an integrated manner. *Intrapersonal aspects* that emerged included the themes of perseverance and commitment, resilience, sense of mastery, courage, positive mindset, and self-regulation. Within the category of *interpersonal aspects*, the themes that emerged were communication skills, teamwork, leadership skills, support, sense of community, and peer relations. *Transpersonal aspects* included appreciation of beauty, spirituality, and continuous growth.

As the objective of this study was to describe participants’ experience with a focus on grit-related responses, only those themes that have been proven to bear a strong resemblance to the definition of grit (shown in bold in Figure 1) will receive explicit attention in the discussion that follows.

## Discussion

### Category 1: Intrapersonal Aspects

The participants’ experiences of being out of their comfort zones at various times during the ABEL program introduced a strong element of self-awareness which, during their reflections, were associated with the development of various intrapersonal strengths. In this respect, one participant reported, “*I learned so much about myself today. Above all I realized that growth happens outside of our comfort zone. That’s exactly what today was: completely out of my comfort zone.”* After a few months, the participants also referred to a profound sense of self-discovery, which was facilitated through the ABEL activities: *“For me, the adventure program tested me a lot and I pushed myself further than I thought I can go*.” This improvement in intrapersonal skills and strengths is supported by numerous studies conducted on the benefits of ABEL programs (Hattie et al., 1997; Cooley et al., 2014; Prince, 2021).

The prominent intrapersonal themes related to grit that emerged from the participants’ reflections were the following.

#### Perseverance and Commitment

It is evident from the participants’ remarks that the challenging activities inherent to the program was a test of their physical endurance. Consequently, these experiences served as a strong metaphor for the discovery and improvement of their own perseverance and commitment, as stated by one of the participants:

*“I realized that although a breather is extremely important, that the best is to bite on your teeth, to stand up and to go on if you are tired. Keep on going… do not stop.”*

After spending three months in their roles as peer helpers, participants’ recollection of the challenging nature of the river rafting and hiking again elicited responses related to their perseverance and commitment in the achievement of goals. In this respect one of them remarked,

*“I have learned—especially at the rapids that sometimes something looks really difficult and you are maybe scared to go through the situation, but… you will do everything in your power to get through it and when you get to the other side you then realize that it wasn’t that bad after all… to just stay motivated and keep on going because you will get through it…”*

Ris (2015) as well as Christensen and Knezak (2014) state that the basic theme of grit is one of persistence in the face of challenge, as seen above. Perhaps the most prominent author on grit, Angela Duckworth, considers perseverance, as well as purposeful and continuous commitment as the essence of this construct (Duckworth et al., 2007). In support of these findings, a study conducted by Ewert and Yoshino (2011) on the influence of a short-term adventure education program on university students’ levels of resilience, the improvement of perseverance was also one of the main themes that emerged when students were asked about their experience.

#### Resilience

During the ABEL program, participants became aware of their capacity to show *resilience* when faced with obstacles. One participant put is as follows:

“*Today I have learned that I am stronger and braver than I thought. At times I was in the water before the difficult parts* (referring to the rapids)*—I was very scared and did not think I could do it, but I did it every time. I realized that I can still function well through tough times.”*

Another participant remarked:

*“The hiking was uphill and with times I wanted to stop and give up, but through renewing my thoughts and to focus on what is important, I was able to pull myself through and with help from above got to the top. Bad and difficult times will follow, and it is okay because after every storm there will be a rainbow.”*

It is clear for the quote above that a number of participants related their ability to show resilience in these challenging circumstances to their religious beliefs:

*“At first, I didn’t want to hike because my chin and arms was burning blisters. But there was just one motivation that made me keep going and that was God. I was beyond my human strength and the power of God carried me. through.”*

This aspect will receive more explicit attention later, under the theme of spirituality. Whatever the source of their resilience, though, the activities that participants engaged in during the ABEL intervention provided an opportunity for many of them to surprise themselves: “*I also learned I have strength for so much, although I underestimate myself easily.”* Perkins-Gough (2013) states, after an interview with Angela Duckworth that grit, amongst other things, involves showing resilience when faced with adversity. The emergence of this theme also confirms the results of Ewert and Yoshino (2011) who reported an improvement of resilience in university students that attended an adventure education program.

#### Courage

Courage refers to the ability to perform an action despite the presence of fear. In turn, when having to persist at a task, grit may be regarded as the courage to overcome the fear of failure (Lucas et al., 2015). Due to the nature of the ABEL experience, participants often had to call upon their *physical* courage to complete challenges successfully. This is defined by Pury and Kowalski (2007) as the ability to take action in the face of physical risk. As is clear from the following quote, team members’ reciprocal support made significant contributions to the courage that they reported:

*“Today’s activities gave me the opportunity to conquer two of my greatest fears: spiders and heights. My teammate found a spider in the water and encouraged me to let the spider walk on my hand (it is amazing what a little encouragement can do!)”*

Participants’ courage was also strongly associated with feelings of accomplishment, as illustrated by the following: *“We also climbed the wall, and I could feel my fear fading as I got higher.”* Rather than avoid the source of fear, one participant alluded to the significant role that the adoption of positive mindset plays in showing courage:

*“What I have learned was to take it on and finish it and not to avoid the problem because it seems impossible. Rather use every moment of fear as an opportunity to learn and get to know another part of yourself and with that to enjoy the moment.”*

As can be seen in Figure 1, the importance of a positive mindset emerged on a number of occasions, which warrants its discussion as a theme on its own.

#### Positive Mindset

It is clear from the discussion of the theme of resilience, that a number of participants achieved this through adopting a positive mindset: *“If things get tough and difficult, I do not just seek the negative. I look for the positive and use it for my next step.”* O’Brien and Lomas (2016) found that adoption of a positive mindset has immense implications for producing successful outcomes in the lives of young people. A few months after the completion of the ABEL intervention, the participants again associated their determination and courage with choosing to adopt a positive frame of mind. They explained that a good attitude and a positive mindset was a crucial factor in following through with the adventure activities, as well as their objectives as peer helpers:

“*Yet again… it is one of those things that comes back to a positive attitude… you can partially choose if you will enjoy something or will not enjoy it. Like when we… it was the last day of hiking when we came to that moment, we didn’t know whether we are going straight on the road or are we going up the mountain… then they told us okay no, we are going up the mountain, with our bags and everything. Then I was just like… “okay, okay, now I must choose, am I going to be negative now or do I go for it?” So, it was just one of those things that you realize again how you approach something will determine how it will be for you in the end. And it was… when we were on top, the view was… it was really worth the effort*.”

Another participant put it as follows, *“I have learned that your brain is your strongest but also your weakest link. Because it is your mind telling you what you cannot do and it sometimes limits you, but it is also your mind that gets you through something in the end.”*

This supports the findings of Pappano (2013), who stated the approach to learning that individuals adopt during their participation in ABEL programs may be an integral part of what they learn. Individuals will be able to embrace challenges and learn from failures when they have a positive mindset, while possessing grit entails working strenuously toward challenges, despite encountering possible setbacks (Duckworth et al., 2007; Reed and Jeremiah, 2017).

#### Self-Regulation

The participants’ realization of the value of a positive mindset was also associated with and built upon their ability of self-regulation. This required the intentional transformation of their thoughts, emotions, and behavior when experiencing demanding situations:

“*… if you started something and you realize closer to the end that it is complicated you can always still find a way to achieve your goal. It may be different than your original plan, but it can also work—thus it is sometimes important to change your approach to something.”*

Self-regulation optimizes the achievement of personal goals through the purposeful alteration of one’s thoughts, emotions, and actions (Zimmerman, 2000). Ivcevic and Brackett (2014) consider grit as a self-regulatory trait. They clarify that to achieve challenging goals requires working hard and the ability to control impulses, and to manage emotions that are related to the pursuit of those goals. The importance of regulating impulses and emotions during the ABEL activities are illustrated well in the following quote from one of the participants’ journals:

*“There were moments where I had to convince myself (my thoughts) to finish the activity. I got so tired physically and I could not regulate it. But if my mind gets tired, I can regulate my thoughts and with that I could overcome the physical shortages.”*

In summary, it is apparent from the participants’ responses that these intrapersonal strengths were not experienced in isolation. When combining the above-mentioned character strengths, the large degree to which the participants’ experiences reflected the essence of grit becomes clear. One participant’s description of her learning experience illustrated the coherence between these themes quite well, “*If you are tired, persist. Look forward to reaching the top. Stay positive. Have courage. See the good in everything.”* Goodwin and Miller (2013) also consider perseverance, tenacity, resilience, stamina, and persistence to be synonyms for grit. Each of these concepts could be independently regarded as a predictor of success, but a greater effect may result from the combination of qualities (Reed and Jeremiah, 2017), which yet again highlights the multidimensionality of grit.

### Category 2: Interpersonal Aspects

Owing to the challenging nature of the ABEL program, the participants often needed to function as close social entities. In addition to participants’ focus on the intrapersonal benefits of the program, they also brought attention to the interpersonal aspects they gained from their participation in ABEL. Interpersonal themes related to grit mainly involved the following:

#### Support

Participants explained that other individuals’ support during the ABEL contributed to their ability to persevere. This made them acutely aware of the value of a support network to get through troubled times. *“I think you should have support from the right people, then you can do anything.”* This awareness was still a strong theme that emerged from their responses during the focus group interviews, three months after completing the ABEL intervention: “*I think community is just really important. I did not realize what the importance of it is in your life, to have a support network… and I only realized it on the camp.”*

The support that participants received from others played an imperative role in their ability to fulfill their roles as peer helpers effectively. Participants also explained that their interpersonal understanding regarding others’ emotions and behavior increased through their participation in ABEL, and that this made them more effective in their role as peer helpers:

*“I think the fact that the camp tested us a lot… up to a point where you felt you do not want to go on… it has taught us empathy, especially with the first years during the R* & *O period. That if they tell us they cannot go on anymore, we know we went through similar experience and how to motivate them… and that they must keep going.”*

This is supported by Neill and Dias (2001) who explain that experiencing challenges with a support network is positively linked to individual growth, and especially effective in building psychological resilience, which—as previously confirmed—is related to grit. The importance of support from group members was echoed in the study by Ewert and Yoshino (2011) that stated that group support was “inevitably important for them [the participants] to overcome adverse conditions and perhaps to better gain a sense of resilience in themselves.”

#### Sense of Community

During the ABEL program, participants also started to experience the value of interdependence and belonging to a group:

*“I think what contributed to my ability to keep on going and to persevere was because I was not alone in it, but that the whole group suffered together and especially that motivated me to go on and finish. I internalized the motivation around me and with that drove myself.”*

After a few months in their role as peer helpers, the participants acknowledged the impact of the ABEL program on the enhancement of their group cohesion. Although they represented different residences, they still experienced a sense of unity, as can be seen in the following:

*“It feels to me as if you sometimes just go about in a professional capacity with each other and then it is that cold friction of “I do not really know you.” But the moment when you get to know each other on such a personal level it helps that you function more optimally in a professional capacity as well.”*

This is confirmed in a study by Rhodes and Martin (2014) who found that an ABEL program improved the sense of community of participants in the workplace as observed by colleagues. Greffrath et al. (2013) also reported that the challenging situations created in ABEL contributes to a sense of community, with all the participants’ experiences occurring in relation with others or with others’ support. Regarding grit, Reed and Jeremiah (2017) ascribes its development not only on what is within us, but on being in the right circumstances with people we can trust, confirming the importance of a sense of community.

It is clear that these interpersonal aspects in combination are imperative to the facilitation of group cohesion, which is an essential component for the development of grit.

### Category 3: Transpersonal/Transcendent Aspects

The participants’ reflections on the ABEL program often went beyond intra- and interpersonal aspects, describing experiences that transcended their current personal functioning and context. Fontana and Slack (1996) defines the term “Transpersonal” as experiences that lie beyond the conditioned ego, recognizing a deeper and more enduring sense of being. Transpersonal psychology is further considered in three parts: psychology beyond ego; psychology of the whole person in an interconnected world and psychology of transformation (Hartelius et al., 2007).

The specific themes that emerged from the data in this regard, and could be related to grit, were the following.

#### Appreciation of Nature

Participants frequently highlighted the fact that their ABEL experience helped them develop an appreciation for a number of aspects that transcended the self: “[My favorite part of today’s adventure activity was…] *the nice chats while we hike and the beautiful view. It makes you realize that there is something bigger out there.*” As described in the previous section, participants experienced a strong sense of interconnectedness with other participants. This connectedness clearly extended to the natural environment in which the ABEL intervention took place, and involved an awareness of how the self and others adapted to the novelty of being in a new and different environment:

*“My favorite part was to listen how everyone experience an activity differently, to walk and listen to everyone’s stories and just to experience nature around me. It is different to be in another environment and to see how everyone adapt to it.”*

Nature has been found to be an essential component in contributing to the facilitation of change (Taylor et al., 2010). In this study, it also emerged as an ideal context for the development of grit. One participant explained their closeness to nature as *“…exhausting, nice, challenging, exciting. It was a day of falling into the river and surviving rapids to sitting peacefully and quietly in the field and realizing how amazing things are.”* Another participant stated, *“Today was challenging, but nice. The moment when I saw the view from the top of the mountain, I forgot about the tough climbing.”* These descriptions match the results of Reed and Jeremiah (2017), who found that grit can be fostered through activities that challenge individuals and, at the same time, encourage them to have fun. This is clearly illustrated by the following:

*“Those rapids! I’ve never felt so completely alive as I did today. I think we all had so much fun on those crocs! My favorite part of it all was being one with nature all day and just spending time in God’s amazing creation!”*

As suggested by the previous quote, participants’ experience of these activities often suggested the presence of a strong spiritual/religious component:

“[My favorite part of today’s adventure activity was…] *to see the view from the top of the mountain. All the plants and small insects that make you realize once again how wonderful everything is created.”*

This aspect will receive more attention in the following section.

#### Spirituality

As mentioned, participants reflected on the realization of their insignificance as being part of something much bigger, which clearly led to experiences of spiritual upliftment during the ABEL intervention:

*“My favorite part of it all was being one with nature all day and just spending time in God’s amazing creation! I encountered God on such a personal level…”*

Their immersion in, and “getting lost” in nature instilled in some participants a newfound sense of self-discovery and knowledge of the self, which all contribute indirectly to the development of grit:

*“Losing yourself in nature and in God is such an awesome experience and I think I have a better idea who I am in Him after today.”*

One participant expressed beautifully how her spiritual experience during the intervention impacted directly on the role she played as peer helper:

*“I also feel that when I speak to someone, I must handle it the way I handled the river rafting. The rapid is the difficult situation, the boat is God or the staff of SCD that will help the person through the situation, and I can only climb into the boat through giving empathy and support and helping steer through it by showing the way, but I cannot be the boat.”*

A number of participants however made a direct link between their ABEL experiences and the development of grit. Expressing the origins of their grit in terms of a religious experience, there was specific reference to the value of their religious beliefs in conquering challenging situations:

*“… I was beyond my human strength and the power of God carried me through… By focusing on God’s grace, I could overcome one of the most difficult challenges.”*

In combination, these results seem to confirm Barton and Miller’s (2015) suggestion that for most people spirituality is the foundation of several positive psychological traits, including grit.

#### Continuous Growth

A significant aspect of the participants’ reflections that illustrates the transcendent nature of their experiences, was their perception of a continuous growth process. During the ABEL program, participants often emphasized that the ABEL component of their training allowed them to step beyond their regular response to a situation, and how this learning experience served as a metaphor which aided in the expansion of their perspectives regarding future situations and different contexts. Its applicability to life in general is powerfully illustrated through the following quotes:

*“My favorite part was when we fell off the boat. Although it was uncomfortable and unpleasant, I have learned a lot from it. Life is difficult and there will be days that we will fall off the boat, but in such times, we should stay focused on the solution and not the problem. The bad things let us appreciate the good.”*

*“Today was challenging, but nice. The moment when I saw the view from the top of the mountain, I forgot about the tough climbing. Life is a climb, but the view is great! Thus, I have learned that it is necessary to just go on, no matter how discouraged you feel. In the end, the results will be worth the effort.”*

Reed and Jeremiah (2017) view future-mindedness as an important aspect of grit that helps a person to persist, even when encountering setbacks and to stay committed until he or she reaches his or her goal. Participants shared their experiences according to their expectations of the R&O program that was lying ahead:

*“Rock climbing*—*there I have noticed the obvious, that you first must get over the difficult part before you can enjoy the best times. For example, R* & *O is the rock climbing and abseiling is the student experience. Both adventure activities of the day reflected the uncertainty and challenges of R* & *O.”*

Three months after the intervention, the participants were able to affirm how these experiences benefited them on a personal level and equipped them for their roles as peer helpers—making the sustainability of the learning experience apparent. Gass (1991) report that the transfer of learning to other areas of the participants’ lives is one of the distinguishing characteristics of ABEL, while Newes and Bandoroff (2004) add that the use of metaphors aids in the process of change and facilitates the transfer of learning. Importantly, as illustrated by the following quote, the participants seemed to continue this process of self-discovery after the program had reached its conclusion:

“*I think what I also learned about myself… especially with the abseiling, is that I was not afraid or anything and I did it quickly, but then I realized I do a lot of things in my life quickly just to get it done with and I do not stop to see what is going on around me… I often do things quickly and then I don’t always see the beauty in the things around me…”*

At the same time, it became clear that the participants had the ability to transfer their learning from one context to another. One participant explained,

*“Like I think… I wish every first year can go on a camp like this, because it really equips you, it changes your perception… you see things in a whole different way… just because of 3 days that you were out of your comfort zone. Because what I think about the whole time what we learned is that there is a comfort zone, a growth zone, and a panic zone and to stay in the comfort zone will get you nowhere, so you should climb into your growth zone and run with it. And that is what I got out of it… to realize to be out of your comfort zone is okay, because that is where the magic happens.”*

It is thus evident from this category of transpersonal/transcendent aspects that, during and after the ABEL program, the participants were able to identify the relevance and inherent benefits of their experiences and the accompanying growth process for future situations. The participants’ feedback demonstrated transcendence of the ABEL experience by means of a continuous process of growth, which equipped them better for a variety of challenging situations. Furthermore, considering all the categories of the findings, the similarities between the themes that emerged from the participants’ reflective diaries during the ABEL program and the themes from the focus group interviews after they had performed their duties as peer helpers for a few months, illustrated the sustainability of the learning experience.

## Conclusion

The aim of this research study was to describe, with a focus on grit-related responses, the subjective experiences of the peer helpers during, as well as after their participation in an adventure-based experiential learning (ABEL) program. The results correspond with previous research that showed ABEL to be a successful intervention in the facilitation of intra- and interpersonal aspects of development (Priest and Gass, 2005; Sheard and Golby, 2006; Sutherland and Stuhr, 2012). In addition, the results indicated growth beyond the self and beyond the present moment to represent a transpersonal/transcendent aspect, which played an important part in the participants’ self-reported experiences. These main categories were prominent in both phases of data collection, confirming the sustainability of the growth process initiated during the intervention.

This group of peer helpers’ experience of participation in an ABEL program not only showed a strong association with the facilitation of grit, but also illustrated how an increased understanding of grit developed during the intervention could be transferred to other areas of life. The participants’ reflections suggested that the challenges faced in an ABEL intervention early in their training program increased their commitment as peer helpers, which, in turn, could enhance the effectiveness of existing student counseling services at universities (Hsi and Chung, 2010). Grit has, however, been found to constitute more than merely work ethic, as it appears to be a character strength that affects all the areas of a person’s life (Reed and Jeremiah, 2017). Therefore, apart from their functioning as peer helpers, increases in grit could also increase the academic performance, professional success, lifetime educational achievement, life goals, and well-being of these participants (Duckworth and Gross, 2014; Eskreis-Winkler et al., 2014).

It is therefore strongly suggested that quantitative follow-up studies are done to empirically investigate the significance of the associations suggested by this exploratory study. An additional recommendation would be to create a participant group through random assignment to include participants from diverse ethnic or language groups for the research population to be more representative of the larger peer helper population in the diverse context of South Africa. Future research should also focus on a longitudinal study on the long-term effects of an ABEL program on peer helpers.

In conclusion, the findings of the current study suggest that grit, which represents a potentially significant strength within the context of the peer helper at South African universities, could be strongly influenced by participation in an ABEL program. Apart from the institutional benefits, the inclusion of an ABEL component in the peer helpers’ training furthermore has the potential to empower the participants themselves in the long term through the transfer of its effect on grit into other life contexts.

## Data Availability Statement

The original contributions presented in the study are included in the article/supplementary material, further inquiries can be directed to the corresponding author.

## Ethics Statement

The studies involving human participants were reviewed and approved by Health Research Ethics Committee (HREC). The patients/participants provided their written informed consent to participate in this study.

## Author Contributions

JP was the promotor and CS and IC was the co-promotors. MP was responsible for the research design and writing of the literature, as well as the process of data collection and data analysis. JP, CS, and IC guided the research, edited, and co-authored this manuscript. All authors contributed to the article and approved the submitted version.

## Conflict of Interest

The authors declare that the research was conducted in the absence of any commercial or financial relationships that could be construed as a potential conflict of interest.

## Publisher’s Note

All claims expressed in this article are solely those of the authors and do not necessarily represent those of their affiliated organizations, or those of the publisher, the editors and the reviewers. Any product that may be evaluated in this article, or claim that may be made by its manufacturer, is not guaranteed or endorsed by the publisher.

## Abbreviations

ABEL: adventure-based experiential learning
NWU: North-West University
R&O: registration and orientation.
